# T_2_ mapping of the heart with a double-inversion radial fast spin-echo method with indirect echo compensation

**DOI:** 10.1186/s12968-015-0108-2

**Published:** 2015-02-25

**Authors:** Tomoe Hagio, Chuan Huang, Aiden Abidov, Jaspreet Singh, Bujji Ainapurapu, Scott Squire, Denise Bruck, Maria I Altbach

**Affiliations:** Biomedical Engineering Graduate Interdisciplinary Program, University of Arizona, Tucson, Arizona USA; Department of Mathematics, University of Arizona, Tucson, Arizona USA; Departments of Radiology and Psychiatry, Stony Brook University, Stony Brook, New York USA; Department of Medicine, University of Arizona, Tucson, Arizona USA; Arizona Sarver Heart Center, University of Arizona, Tucson, Arizona USA; Department of Medical Imaging, University of Arizona, Tucson, Arizona USA

**Keywords:** Cardiovascular magnetic resonance, Myocarditis, Edema, T2, Mapping, Radial, FSE, Indirect echo

## Abstract

**Background:**

The abnormal signal intensity in cardiac T_2_-weighted images is associated with various pathologies including myocardial edema. However, the assessment of pathologies based on signal intensity is affected by the acquisition parameters and the sensitivities of the receiver coils. T_2_ mapping has been proposed to overcome limitations of T_2_-weighted imaging, but most methods are limited in spatial and/or temporal resolution. Here we present and evaluate a double inversion recovery radial fast spin-echo (DIR-RADFSE) technique that yields data with high spatiotemporal resolution for cardiac T_2_ mapping.

**Methods:**

DIR-RADFSE data were collected at 1.5 T on phantoms and subjects with echo train length (ETL) = 16, receiver bandwidth (BW) = ±32 kHz, TR = 1RR, matrix size = 256 × 256. Since only 16 views per echo time (TE) are collected, two algorithms designed to reconstruct highly undersampled radial data were used to generate images for 16 time points: the Echo-Sharing (ES) and the CUrve Reconstruction via pca-based Linearization with Indirect Echo compensation (CURLIE) algorithm. T_2_ maps were generated via least-squares fitting or the Slice-resolved Extended Phase Graph (SEPG) model fitting. The CURLIE-SEPG algorithm accounts for the effect of indirect echoes. The algorithms were compared based on reproducibility, using Bland-Altman analysis on data from 7 healthy volunteers, and T_2_ accuracy (against a single-echo spin-echo technique) using phantoms.

**Results:**

Both reconstruction algorithms generated in vivo images with high spatiotemporal resolution and showed good reproducibility. Mean T_2_ difference between repeated measures and the coefficient of repeatability were 0.58 ms and 2.97 for ES and 0.09 ms and 4.85 for CURLIE-SEPG. In vivo T_2_ estimates from ES were higher than those from CURLIE-SEPG. In phantoms, CURLIE-SEPG yielded more accurate T_2_s compared to reference values (error was 7.5-13.9% for ES and 0.6-2.1% for CURLIE-SEPG), consistent with the fact that CURLIE-SEPG compensates for the effects of indirect echoes. The potential of T_2_ mapping with CURLIE-SEPG is demonstrated in two subjects with known heart disease. Elevated T_2_ values were observed in areas of suspected pathology.

**Conclusions:**

DIR-RADFSE yielded TE images with high spatiotemporal resolution. Two algorithms for generating T_2_ maps from highly undersampled data were evaluated in terms of accuracy and reproducibility. Results showed that CURLIE-SEPG yields T_2_ estimates that are reproducible and more accurate than ES.

## Background

T_2_-weighted imaging is an important technique in Cardiovascular Magnetic Resonance (CMR) and it has been used for the diagnosis of a series of pathologies [1-10]. Because inflammation in tissue leads to T_2_ contrast changes, T_2_-weighted imaging can be used to detect myocardial edema [9]. Currently, the most frequently used technique to look at edema in the heart is the triple inversion recovery prepared sequence (triple IR), which yields black-blood images with fat suppression [10]. The images are interpreted by looking at high signal intensity regions within the myocardium that are indicative of water accumulation. A drawback of the method is that the contrast between diseased and normal myocardium is dependent on the choice of parameters, e.g., TE. Furthermore, the signal intensity modulation caused by the use of multiple receivers (i.e., coil sensitivities) makes it more challenging to distinguish edematous areas from healthy myocardium.

T_2_ mapping of the heart has been proposed as an alternative for diagnosing myocardial edema [11-14]. Kim, et al. [11] implemented a breath-hold ECG-triggered double inversion recovery (DIR) multi-echo fast spin-echo (FSE) pulse sequence as a black-blood technique to quantify R_2_ (1/T_2_) in the myocardium. The method is fast and has high temporal resolution (data for 10 TE time points are acquired within a breath hold) but low in-plane spatial resolution (acquisition matrix size = 128 × 72) due to the time constraints imposed by the breath hold. Bright-blood techniques such as the T_2_-prepared steady-state free-precession (T_2_ prep-SSFP) methods [12-14] have also been proposed for T_2_ mapping of the heart for breath-hold [12,13] and free-breathing [14] acquisitions. The technique yielded images with better spatial resolution (acquisition matrix size = 130 × 160) than the Cartesian DIR-FSE method but with lower temporal resolution (only 2 to 3 TE time points). In the T_2_ prep-SSFP pulse sequence the TE images are collected sequentially, which can introduce misregistration between time points [13,14].

In this work we present and evaluate a double inversion recovery radial fast spin-echo (DIR-RADFSE) technique [15,16] for T_2_ mapping of the heart. Because in radial acquisitions each radial line goes though the center of k-space, a single DIR-RADFSE k-space data set can be divided into partial sets from which images at different TEs (number of TEs = ETL) can be reconstructed [17]. In a typical setup for CMR applications we collect data with high temporal resolution (16 TE time points) and since data for all TEs are acquired in each TR period the effects of misregistration between TE sets are minimized. Higher spatial resolution can also be achieved because the spatial resolution is primarily determined by the number of readout points in radial sampling. Radial k-space trajectories are intrinsically more robust against motion artifacts compared to the conventional Cartesian k-space scanning [18,19], a clear advantage for T_2_-weighted imaging of the heart. To limit the scan time to a breath hold we only collect a limited number of radial views per TE (typically 16 views) thus, each TE data set is highly undersampled. Images from highly undersampled k-space data can be reconstructed with an echo sharing (ES) algorithm [17] or a model-based algorithm we recently developed: *CUrve Reconstruction via pca-based Linearization with Indirect Echo compensation* (CURLIE) algorithm [20]. CURLIE takes into account the effect of indirect echoes (e.g., stimulated echoes) that are present in multi-echo spin-echo acquisitions [20]. The DIR-RADFSE technique is evaluated here in phantoms and in vivo.

## Methods

All human studies were performed under informed consent with a protocol approved by the University of Arizona Institutional Review Board.

**Figure 1:**
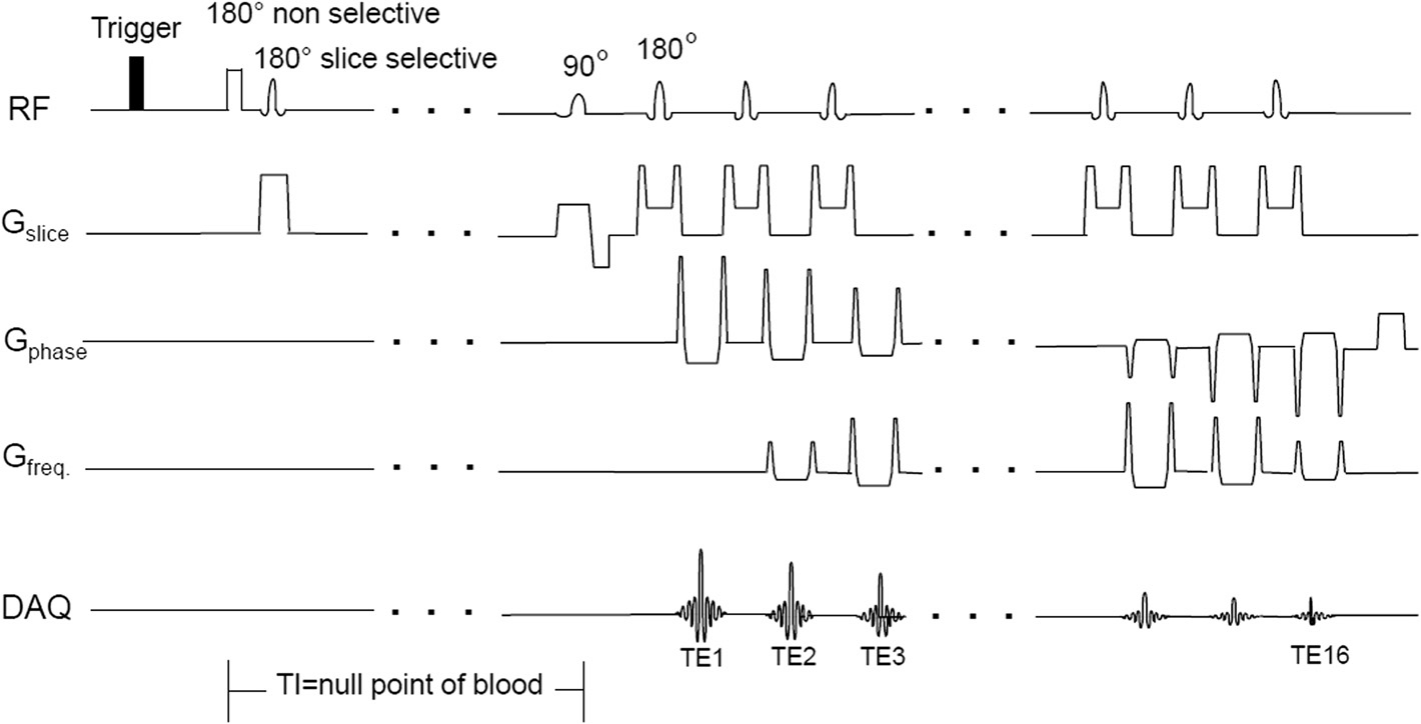
**DIR-RADFSE pulse sequence diagram.** The ECG or peripheral gating signal triggers a double inversion recovery preparation period to null the signal from flowing blood. After the null point of blood, radial FSE data are collected with and ETL = 16.

All data were acquired on a 1.5 T Signa HDxt GE MR scanner (General Electric Healthcare, Milwaukee, WI) using the DIR-RADFSE pulse sequence [15,16]. As shown in Figure 1, the ECG or peripheral gating (PG) signal triggers a DIR preparation period to null the signal from flowing blood [21]. After the null point of blood, data are collected using a radial FSE acquisition scheme. The angular order of radial views is chosen to minimize artifacts from T_2_ decay and motion as well as to provide good k-space coverage for each TE data set [22].

### In vivo imaging

Data were acquired in a breath hold (16-22 s depending on heart rate) using an 8-channel phased-array receiver coil with ETL = 16, for a total of 256 radial views with 256 readout points per view. The total number of radial views per TE was 16 (~4% sampling relative to the Nyquist condition). Other parameters were: TR = 1RR, slice thickness = 8 mm, field-of-view (FOV) = 48 × 48 cm^2^, and BW = ± 31.25 kHz. In this work we used PG triggering because it naturally adds ~200 ms to the time between the R-wave and the trigger pulse. When adding the PG delay to the inversion time (TI) for nulling blood (300-400 ms for heart rates in the range of 60-80 bpm and TR = 1RR), the FSE acquisition is naturally timed to start in diastole. Saturation bands were placed within the TI period, prior to data acquisition, to suppress unwanted signal from structures within and outside the FOV. Chemical-shift selective saturation was used for fat suppression. Patients were scanned with the DIR-RADFSE pulse sequence as part of a clinical CMR exam. For these subjects Late Gadolinium Enhancement (LGE) images were acquired during the systolic phase of the cardiac cycle, 10 to 15 minutes after the intravenous injection of MultiHance (gadobenate dimeglumine, Bracco Diagnostics Inc., USA).

### Phantom study

Phantoms with 6 different T_2_ values were prepared using MnCl_2_ solutions (50 μM, 75 μM, and 150 μM) or agar gels at different concentrations (0.6%, 1.2%, and 2.0%) mixed with 1.5 mM NiCl_2_ (to adjust the T_1_ to ~ 900 ms). The phantoms covered T_2_ values in the range of 38-170 ms.

The phantoms were imaged with the DIR-RADFSE pulse sequence with the same parameters described above except for FOV = 24 × 24 cm^2^; the heart rate was simulated to 60 bpm so that TR = 1 s. T_2_ mapping was performed using the ES and the CURLIE-SEPG algorithms, as described below. A region of interest (ROI) was manually drawn to encompass all the pixels within the phantom and the mean T_2_ was obtained for each ROI. T_2_ measurements were also carried out with a Cartesian single-echo spin-echo pulse sequence with TE = 9, 18, 27, and 36 ms, TR = 3 seconds, and matrix size = 128 × 64. The latter method was used as a reference for T_2_ estimation without the effect of indirect echoes. The reference T_2_ values were calculated by fitting the TE images to a single exponential signal model using least-squares fitting.

### Image reconstruction and T_2_ mapping

**Figure 2:**
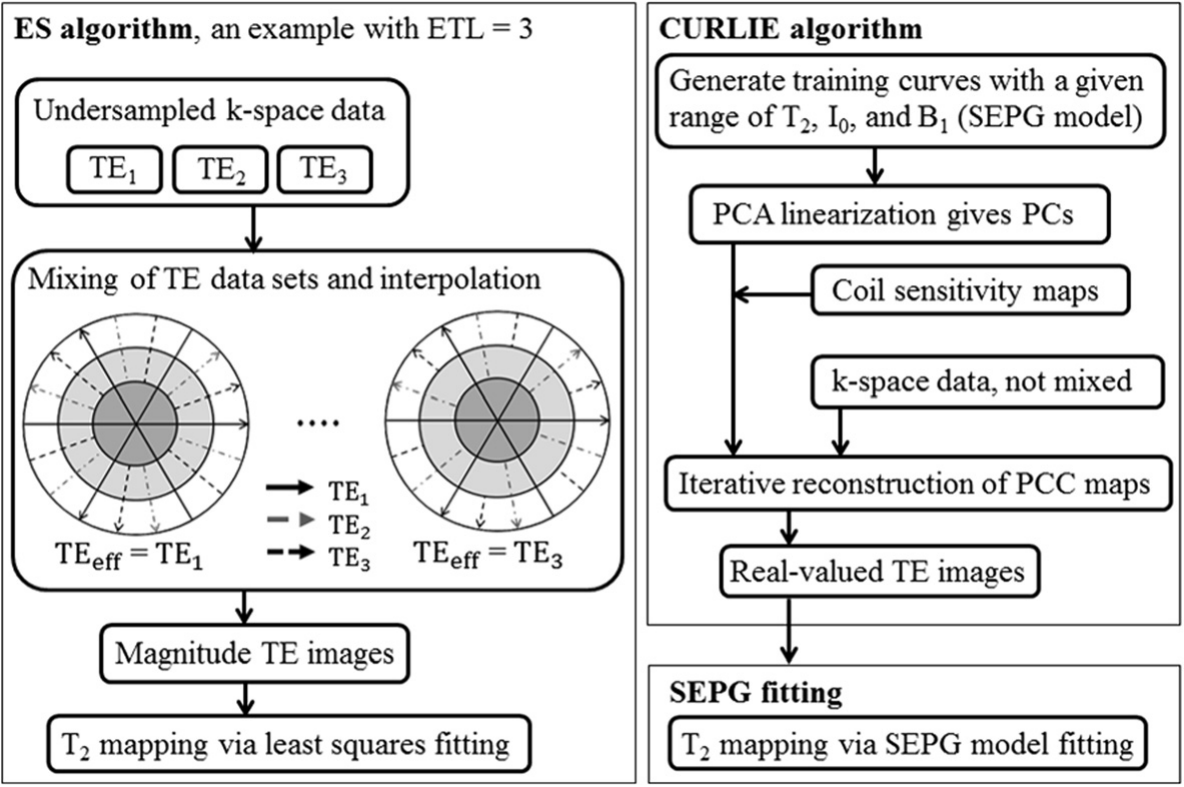
**Flow charts of the ES and the CURLIE algorithms.** The left panel shows a diagram for the generation of TE images with the ES algorithm by mixing TE data in k-space. For simplicity an ETL = 3 is used in the diagram (compared with ETL = 16 used in this paper). T_2_ maps are generated after a least-squares fitting of the magnitude TE images. The right panel outlines the main steps for the generation of the TE images based on the CURLIE algorithm. T_2_ maps are generated after SEPG fitting of the complex TE images.

All algorithms were implemented in Matlab (MathWorks, Natick, MA). Figure 2 shows a flow chart for the ES and CURLIE algorithms used for the reconstruction of TE images from highly undersampled data. As indicated in the figure, the ES approach mixes radial views acquired at different TEs to form k-space data sets weighted to the TE of the data in the center. The TE k-space data sets are used to reconstruct magnitude images at different effective TEs (TE_eff_) for each receiver coil using filtered back-projection. The sum-of-squares of the individual receiver coil images is used to obtain the TE images that are used in the T_2_ estimation. The TE images are fit to a single exponential signal model using least-squares fitting to obtain the T_2_ map.

CURLIE is a model-based reconstruction algorithm where the TE data sets are not mixed but used in an iterative manner by fitting the expected signal model to the acquired TE data [20]. A general frame work for model-based reconstructions for FSE data is given below:

1 $$ \widehat{\boldsymbol{\theta}}= argmi{n}_{\boldsymbol{\theta}}\left\{{\sum}_n\left\Vert F{T}_n\left({S}_n\left(\boldsymbol{\theta}, T{E}_n\right)\right)-{\boldsymbol{K}}_n\left\Vert {}^2\right.\right.\right\}, $$

where *FT*(⋅) is the forward Fourier transform and *S*(^**.**^) describes the model of the signal in the image domain according to a set of parameters ***θ*** and the TE time points. ***K***_*n*_ are the acquired k-space data at *TE*_*n*_, the TE at the *n*^th^ SE point. Equation (1) minimizes the difference between the acquired k-space data and the signal model by iterating over the values of *θ*_*.*_

To account for the effect of indirect echoes in the signal model we use the Slice-resolved Extended Phase Graph (SEPG) model developed by Lebel and Wilman [23]. SEPG incorporates the effects of flip angle imperfections through a known slice profile (e.g., along *z*). Given the flip angles of the excitation pulse (*α*_*0*_(*z*)) and refocusing pulses (*α*_*n*_(*z*)*; n* = 1, …, ETL), the sensitivity of the transmit *B*_1_ field, and the *I*_o_, *T*_2_, and *T*_1_, the signal intensity of the *n*^th^ echo point can be represented by:

2 $$ {S}_n={I}_0{\displaystyle \int }EPG\left({T}_1,{T}_2,{B}_1,{\alpha}_0(z),\cdots, {\alpha}_n(z),n\right)\ dz $$

We have previously shown that non-linear equations (such as (2)) make model-based reconstruction unstable. To overcome this problem we developed a principal-component based reconstruction, where a linear approximation to the signal model based on principal components (PCs) is used [24]. Thus, Eq. (1) can be reformulated as:

3 $$ \widehat{\boldsymbol{M}}= argmi{n}_{\boldsymbol{M}}\left\{{\displaystyle {\sum}_{n=1}^L{\left|\left|FT\left(\boldsymbol{M}{\widehat{\boldsymbol{P}}}_n^T\right)-{\boldsymbol{K}}_n\right|\right|}^2}\right\}, $$

where $\widehat{\boldsymbol{P}}$ is the matrix consisting of the vectors of PCs, generated by singular value decomposition from a set of T_2_ training curves. ***M*** is the vector of the PC coefficients and is obtained using a conjugate gradient minimization algorithm. TE images are then generated from the matrix of PC coefficients, $\widehat{\boldsymbol{M}}$, and $\widehat{\boldsymbol{P}}$_._

The algorithm used in this work, incorporates complex coil sensitivities (*C*_*j*_) and a penalty term that exploits the spatial compressibility of the PC coefficient maps according to the compressed sensing theory [25] into Eq. (3):

4 $$ \widehat{\boldsymbol{M}}= argmi{n}_{\boldsymbol{M}}\left\{{\displaystyle {\sum}_{j=1}^{\# coils}{\displaystyle {\sum}_{n=1}^L\left\Vert FT\right.}}\;\left({C}_j\boldsymbol{M}{\widehat{\boldsymbol{P}}}_n^T\right)-{\boldsymbol{K}}_{j,n}{\left\Vert\;\right.}^2+{\displaystyle {\sum}_i{\lambda}_i} Penalt{y}_i\left(\boldsymbol{M}\right)\right\}. $$

The steps of the CURLIE-SEPG algorithm are summarized in Figure 2. The training curves for obtaining the PCs were derived using the SEPG model for T_2_ = 30-300 ms (increment = 1 ms) and B_1_ = 0.8-1.2 (increment = 0.05). Since SEPG fitting is rather insensitive to T_1_ [20,23] we fixed T_1_ = 1000 ms based on the literature values for myocardium [26]. The number of PCs used was 6. The penalty term in (4) consisted of the 1-norms of the wavelet transform (Daubechies 4, code obtained from http://www-stat.stanford.edu/~wavelab) and the total variation of the PC coefficient maps. A weight of 0.005 was used for the penalty terms. The coil sensitivities were calculated by dividing the complex images for each coil by the sum-of-squares of the images for all coils. The complex coil images used for the coil sensitivity estimation were obtained by combining k-space data from all TEs (i.e., data from 256 radial views) followed by filtered-back projection. The coil sensitivity maps were smoothed to reduce noise. Once the TE images were generated from $\widehat{\boldsymbol{M}}$ and $\widehat{\boldsymbol{P}}$, T_2_ maps were obtained via SEPG fitting using Eq. (2).

### Reproducibility study

T_2_ estimation was compared among reconstruction algorithms using the mean T_2_ values of a ROI manually drawn on the left ventricle (LV). In the reproducibility study, mean T_2_ estimates from each experiment were compared using the Bland-Altman analysis.

## Results

### T_2_ Estimation with DIR-RADFSE

**Figure 3:**
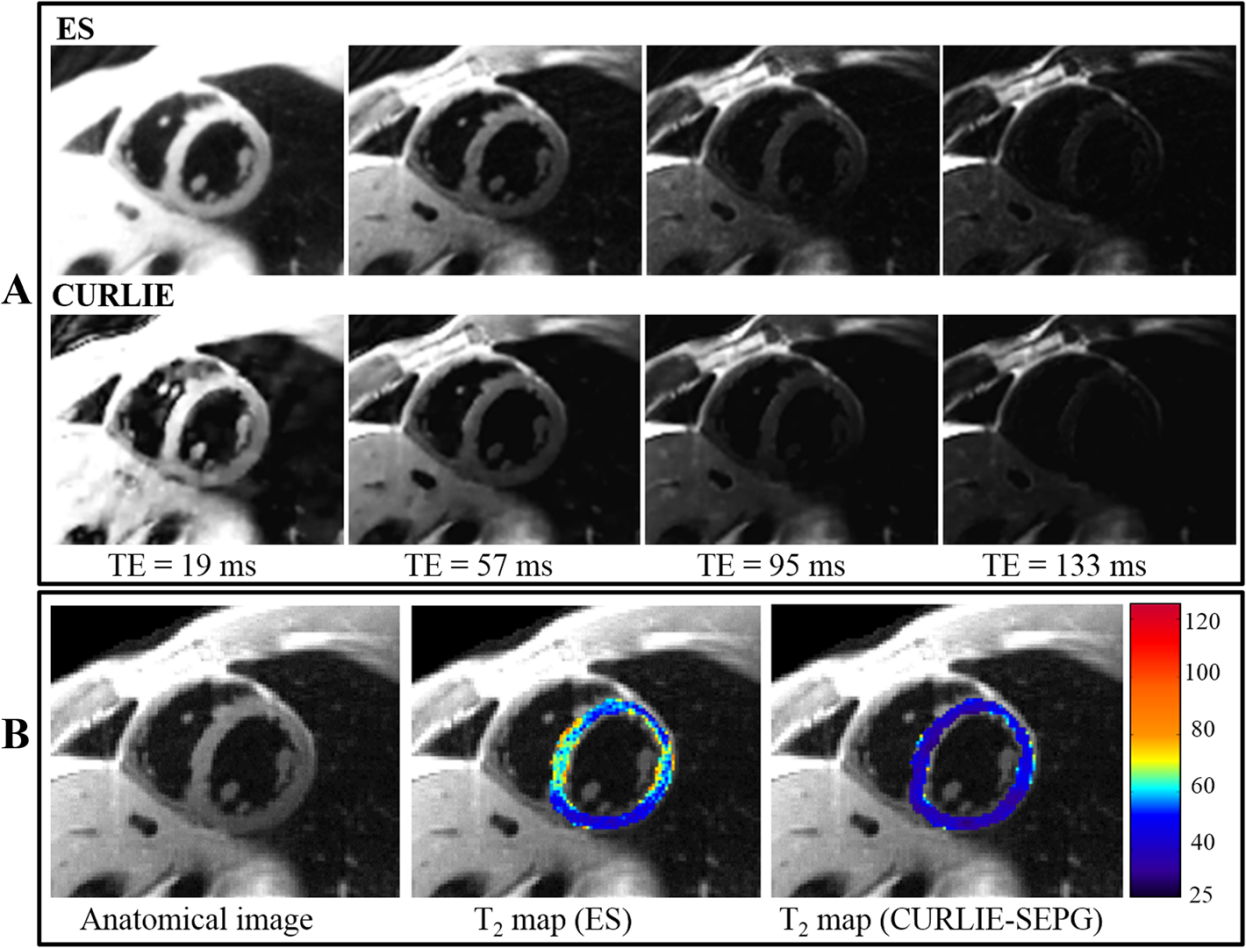
**Images of a healthy volunteer obtained with DIR-RADFSE. (A)** The TE images are reconstructed from undersampled data sets (16 radial views per TE) using the ES and the CURLIE algorithms. **(B)** The anatomical image, reconstructed by filtered back-projection using the full k-space data set (all 256 radial views), and a colorized T_2_ map of the left ventricle myocardium overlaid on the anatomical image are displayed for both algorithms. The anatomical image, TE images and T_2_ maps are generated from the same k-space data (acquired in a single breath hold).

Images reconstructed from DIR-RADFSE data acquired in a single breath hold for a normal volunteer are shown in Figure 3. Figure 3A shows 4 (out of 16) images at different TEs obtained from the undersampled data sets (16 radial views per TE) using the ES and the CURLIE reconstruction algorithms. Figure 3B (left) shows the image generated from the full k-space radial data set (256 radial views). This type of image (referred throughout this paper as the anatomical image) has a T_2_-weighted contrast comparable to a TE of 57 ms. Figure 3B also shows the colorized T_2_ maps of the LV myocardium overlaid on the anatomical image for both ES and CURLIE-SEPG. Note that the ES reconstruction yields higher T_2_ values than the CURLIE-SEPG algorithm. This can also be observed by the slightly slower signal decay of the myocardium in the ES TE images compared to the CURLIE reconstruction.

T_2_ estimation on phantoms (Table 1) showed the same trend. Note that T_2_ estimates are higher with the ES reconstruction than with CURLIE-SEPG. The latter were closer to the reference T_2_ values obtained from a single-echo spin-echo experiment. The T_2_ estimation error was 0.6-2.1% for CURLIE-SEPG and 7.5-13.9% for ES. The standard deviations were also smaller with CURLIE-SEPG than ES.

**Table 1:** **Mean and standard deviation (stdev) of T**_**2**_ **estimates on phantoms with different T**_**2**_ **values**

**Gold standard**	**ES**			**CURLIE-SEPG**		
**T**_**2**_ **(ms)**	**T**_**2**_ **mean (ms)**	**T**_**2**_ **stdev (ms)**	**% error**	**T**_**2**_ **mean (ms)**	**T**_**2**_ **stdev (ms)**	**% error**
38	43.28	4.74	13.89	38.80	1.24	2.11
55	60.71	5.10	10.38	55.79	2.72	1.44
75	81.16	6.67	8.21	75.97	2.93	1.29
78	85.00	6.83	8.97	78.46	2.49	0.59
112	120.88	9.41	7.93	112.73	4.64	0.65
170	182.69	9.65	7.46	171.60	6.12	0.94

The reference T_2_ values were obtained from a single-echo spin-echo experiment.

### Reproducibility study

**Figure 4:**
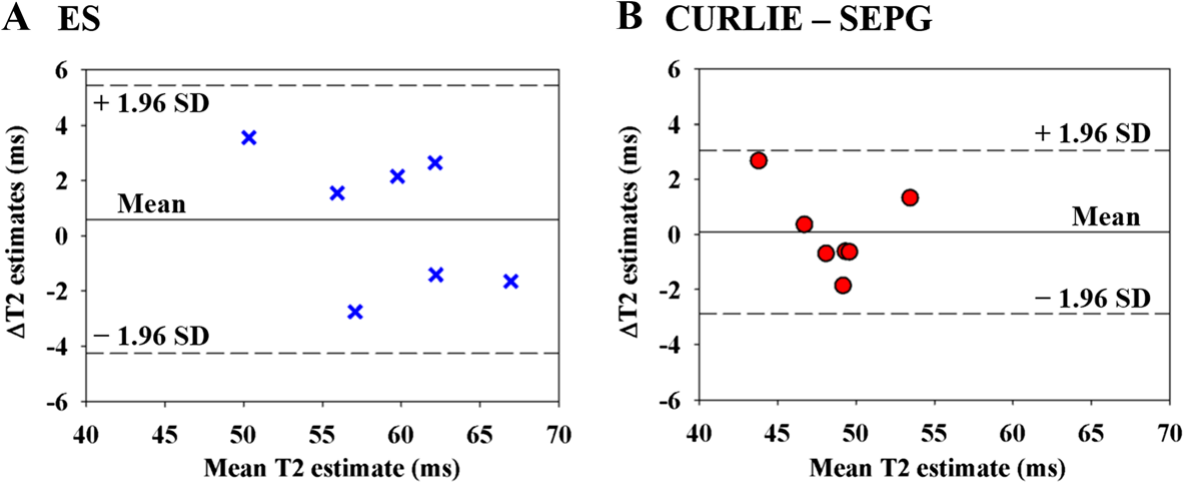
**Bland-Altman plots for the (A) ES and (B) CURLIE-SEPG reconstructions.** Each point in the figure shows the mean T_2_ estimates (x-axis) and the difference in the pair of T_2_ estimates (y-axis). The central horizontal lines in each plot are the mean of the differences in the pairs of T_2_ estimates. The outer dotted horizontal lines mark the lower/upper limits of agreement, calculated as: mean T_2_ difference ± 1.96 × standard deviation of T_2_ differences.

To evaluate the reproducibility of T_2_ mapping with DIR-RADFSE we imaged 7 healthy volunteers; each subject was imaged twice during the same imaging session. T_2_ maps were generated with the ES and the CURLIE-SEPG algorithms to test the effect of reconstruction on the reproducibility of T_2_ mapping. The Bland-Altman plots (Figure 4) and analysis (Table 2) show high agreement between T_2_ values from repeated measures for both reconstruction algorithms. The mean T_2_ difference was slightly lower with CURLIE-SEPG (0.09 ms) than with ES (0.58 ms). The coefficient of repeatability were 2.97 for CURLIE-SEPG and 4.85 for ES, also indicating slightly better reproducibility with CURLIE-SEPG.

**Table 2:** **Bland-Altman analysis**

**Reconstruction algorithm**	**Limits of agreement (ms)**			**Coefficient of repeatability** ^**a**^
	**Mean ∆T**_**2**_	**Lower limit**	**Upper limit**	
**ES**	0.58	−4.27	5.43	4.85
**CURLIE-SEPG**	0.09	−2.88	3.06	2.97

^a^Coefficient of repeatability is defined as 1.96 times the standard deviation of the T_2_ differences between the two measurements. Thus, a lower coefficient of repeatability indicates higher reproducibility.

**Figure 5:**
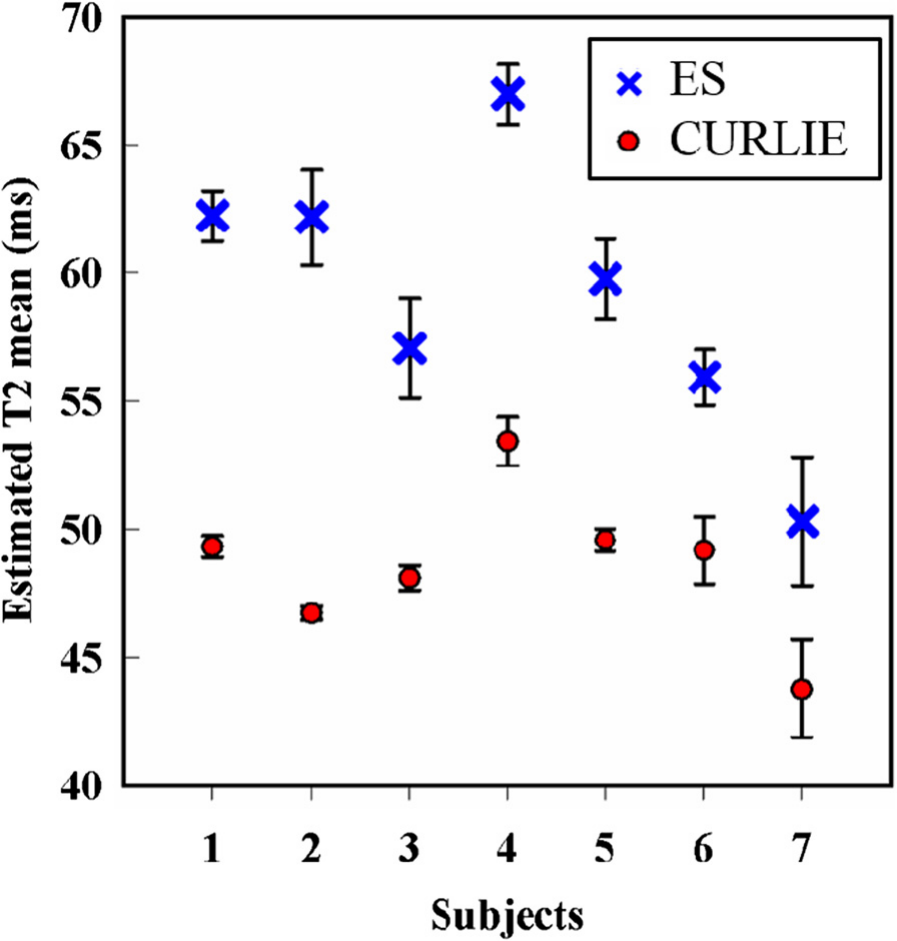
**Plot of mean T**_**2**_ **values of the left ventricle myocardium in healthy subjects.** Data for 7 healthy subjects are shown for T_2_ estimates obtained with the ES and the CURLIE-SEPG algorithms. In all subjects, T_2_ values are higher with the ES algorithm compared to the CURLIE-SEPG algorithm.

In agreement with the results shown above, the T_2_ estimates obtained with ES were higher than those with CURLIE-SEPG for all 7 volunteers, as shown in Figure 5.

### T_2_ mapping with DIR-RADFSE and CURLIE-SEPG in clinical CMR

**Figure 6:**
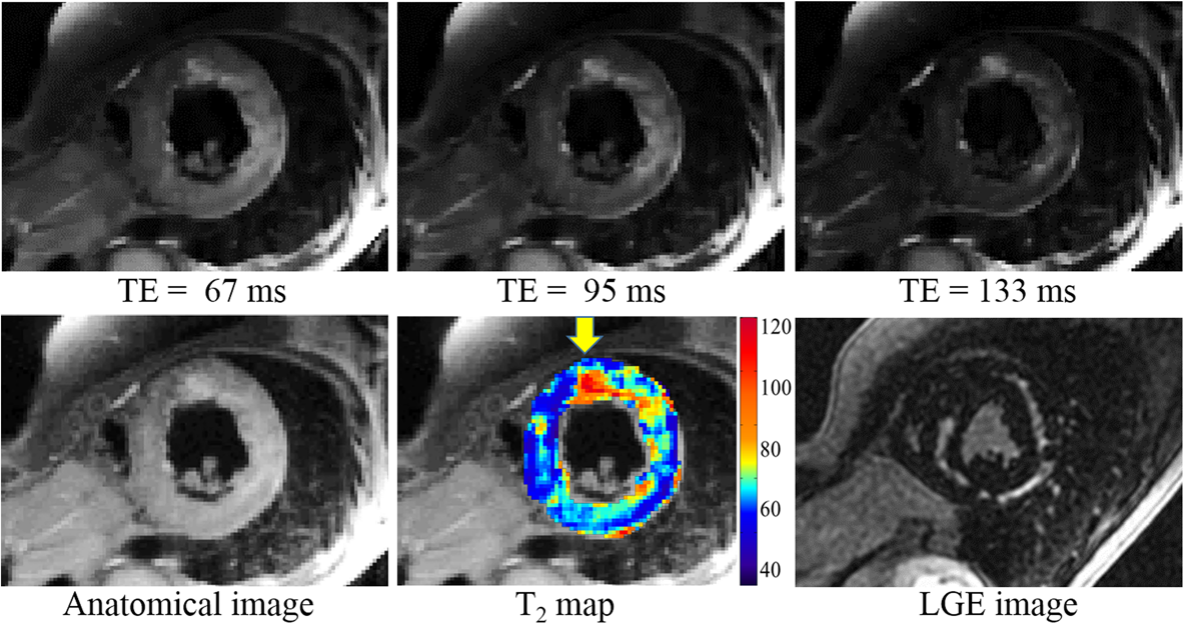
**Images of a subject diagnosed with hypertrophic cardiomyopathy.** (Top) Three out of the 16 TE images reconstructed from undersampled data sets (16 radial views per TE) using CURLIE. (Bottom) The anatomical image, reconstructed by filtered back-projection using the full k-space data set (all 256 radial views), is shown on the lower left panel. The colorized T_2_ map of the left ventricle overlaid onto the anatomical image is displayed in the lower middle panel for CURLIE-SEPG. The LGE image is shown in the lower right panel.

An example of DIR-RADFSE in a subject diagnosed with hypertrophic cardiomyopathy and ventricular ectopy is shown in Figure 6. The top panel shows 3 out of the 16 TE images reconstructed from undersampled TE data (16 radial views per TE) using CURLIE. The bottom panel shows the anatomical image together with the colorized T_2_ map of the LV overlaid on the anatomical image. The LGE image is also shown in the figure. There are areas of high T_2_ signal in the lateral and inferior LV wall and the RV insertion points which correspond to areas of fibrosis seen on the LGE image. In addition, the TE images and T_2_ map reveal a focal area of high T_2_ (T_2_ > 120 ms) in the anterior and antero-septal segment (as indicated by the arrow) with no matching area of fibrosis on the LGE image, suggesting the presence of edema. This is consistent with recent reports [1,27-30], where T_2_ imaging was found to be a more sensitive tool of edematous inflammatory processes than LGE.

**Figure 7:**
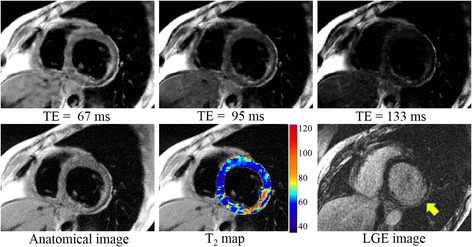
**Images of a patient with myocardial infarct scar.** (Top) Three out of the 16 TE images reconstructed from undersampled data sets (16 radial views per TE) using CURLIE. (Bottom) The anatomical image, reconstructed by filtered back-projection using the full k-space data set (all 256 radial views), is shown on the lower left panel. The colorized T_2_ map of the left ventricle overlaid onto the anatomical image is displayed in the lower middle panel for CURLIE-SEPG. The LGE image is shown in the lower right panel.

Another case for a subject with a history of cardiomyopathy, coronary artery disease, and a myocardial infarction incidence, is shown in Figure 7. The infarct region is typically characterized by a thinning of the myocardial wall as well as by the presence of sub-endocardial and/or transmural scar. In this patient, predominantly sub-endocardial scar tissue can be seen as an area of bright signal in the LGE image in the inferior and infero-lateral wall (arrow). The clinical findings for this patient did not indicate the presence of edema. The DIR-RADFSE images are in concordance with the LGE images showing the thinned myocardial wall and higher T_2_ values around the region corresponding to scarred tissue in the LGE image. The average T_2_ around the scarred region (arrow) was ~89 ms. In contrast, the rest of the myocardium had the average T_2_ of ~53 ms. The slight increase in T_2_ in this case might be due to fluid in the extracellular space within the scar.

## Discussion

In this work, we introduced and evaluated a black-blood radial FSE imaging technique, DIR-RADFSE, for T_2_ mapping of the heart. Using the DIR-RADFSE pulse sequence combined with algorithms tailored to reconstruct highly undersampled data we have shown that we can obtain as many as 16 TE images with high spatial resolution from data acquired in a single breath hold. Since all TE points are collected within each TR period, misregistration between data sets is minimized, and T_2_ mapping can be performed voxel-wise without the need of image registration. The radial acquisition has also an advantage over Cartesian trajectories for CMR applications due to its inherent robustness to motion [18,19].

The limited number of k-space lines available at each TE requires reconstruction algorithms that can compensate for the effects of undersampling. The algorithms evaluated in this work, ES and CURLIE-SEPG, have been designed to reconstruct TE images from undersampled radial data. In sets of data acquired on 7 volunteers, both algorithms have shown to yield highly reproducible T_2_ maps. However, the estimated T_2_ values were higher with the ES algorithm than with CURLIE-SEPG.

The T_2_ overestimation by ES comes from several factors. Indirect echoes, which are a consequence of imperfections associated with the refocusing pulses in multiple echo pulse sequences, are known to lead to T_2_ overestimation. The major difference and the benefit of the new algorithm, CURLIE combined with SEPG fitting, is that it incorporates the effect of indirect echoes in the signal model thus, reducing T_2_ overestimation.

The magnitude reconstruction used to obtain the TE images in the ES algorithm is also a source of T_2_ overestimation. Taking the magnitude of the data causes the noise distribution to become non-Gaussian (i.e. strictly positive) for data with low signal-to-noise ratio (SNR) [31]. Thus, for data at the latter TEs (where the SNR may be compromised) the magnitude operation artificially increases the signal intensity of these time points, which in turn yields higher T_2_ values. This is not specific to the ES algorithm but to every algorithm that is based on a magnitude reconstruction (note that we have used a magnitude operation for the reference spin-echo data, however the SNR of the reference scan was higher because the data was fully sampled). In contrast, the model-based CURLIE algorithm does not suffer from the problems associated with a magnitude reconstruction because the data fitting is done in k-space using complex data [20,24].

TE mixing in the ES algorithm can also cause T_2_ overestimation [17]. As can be seen in Figure 2, TE mixing increases from the center to the outer part of k-space (the high spatial frequency region). This affects the T_2_ estimation of objects with high spatial frequency content as the edges of the myocardial wall.

An advantage of the ES reconstruction is its reconstruction speed compared to the CURLIE-SEPG method, which is based on an iterative and more computationally expensive algorithm. Improvements of the ES reconstruction, such as incorporating a thicker refocusing slice [32] or using a spoiler gradient editing method [33-35] for reducing the effects of indirect echoes as well as implementing a reconstruction based on the fitting of complex data, can alleviate T_2_ overestimation.

DIR-RADFSE is a black-blood imaging method which relies on the suppression of flowing blood. As reported before, a drawback of black-blood T_2_-weighted images (e.g., the triple IR method) is that the high signal intensity of stagnant (unsuppressed) blood makes it difficult to differentiate edema from blood stasis at the infarct-tissue border [1,36]. The advantage over the conventional triple IR method is that DIR-RADFSE yields T_2_ maps, in addition to the T_2_-weighted images, allowing for a “quantitative” assessment of the myocardium. Thus, with DIR-RADFSE we expect that the signal from blood stasis should be differentiated from the myocardium based on differences in T_2_ values provided that the spatial resolution is adequate to resolve the endocardial region from the blood pool. Another advantage of using T_2_ values to characterize changes in the myocardium instead of differences in signal intensity (as done with conventional triple IR methods) is the insensitivity of T_2_ maps to coil sensitivities.

## Conclusions

In this work, we present results of cardiac T_2_ mapping using the DIR-RADFSE technique. DIR-RADFSE yields TE images with high spatial and temporal resolution from data acquired in a single breath hold. Since the acquired data per TE time point are highly undersampled, two algorithms were evaluated for image reconstruction and T_2_ mapping: the echo sharing (ES) algorithm and the model-based CURLIE-SEPG algorithm. Although both algorithms yielded reproducible T_2_ maps, CURLIE-SEPG yielded T_2_ estimates that are closer to a reference standard, the single-echo spin-echo method. This is consistent with the fact that CURLIE-SEPG compensates for the effect of indirect echoes. The technique can have a significant impact in the detection of pathologies characterized by abnormal T_2_ values.
